# The Influence of GLP-1 Agonists on Human Mesenchymal Stem Cells: A Systematic Review

**DOI:** 10.1007/s12015-025-11002-7

**Published:** 2025-10-23

**Authors:** Luisa Weber, Maryam Hashemnia Sharbabaki, Benedikt Fuchs, Paolo Alberton, Riccardo Giunta, Sinan Mert, Nikolaus Thierfelder

**Affiliations:** Division of Hand, Plastic and Aesthetic Surgery, LMU University Hospital, Munich, Germany

**Keywords:** Glucagon-like-peptide-1, Glucagon-like-peptide-1 receptor, Human mesenchymal stem cells, Liraglutide, Semaglutide, Exendin-4, Tirzepatide, Regenerative medicine

## Abstract

**Background:**

Glucagon-like peptide-1 receptor agonists, originally developed for managing type 2 diabetes, have gained attention for their weight-reducing and broader biological effects. Among these, their influence on human mesenchymal stem cells remains underexplored, despite the critical role of mesenchymal stem cells in tissue regeneration and secretion of bioactive factors.

**Methods:**

This systematic review followed the Preferred Reporting Items for Systematic Reviews and Meta-Analyses (PRISMA) guidelines to identify and evaluate in vitro studies investigating the effects of glucagon-like peptide-1 receptor agonists and their analogues on human mesenchymal stem cell functions, including proliferation, differentiation, signaling, apoptosis, and tissue-specific applications. Risk of bias was assessed using an adapted Quality Assessment Tool for In Vitro Studies (QUIN) tool.

**Results:**

Thirty-eight eligible studies were identified. Glucagon-like-peptide-1 receptor agonist, like native glucagon-like peptide-1, Exendin-4, and Liraglutide, exert context-, dose-, and timing-dependent effects on human mesenchymal stem cells. They modulate proliferation and overall promote osteogenesis while inhibiting adipogenesis. Key pathways, including Wnt/β-catenin, bone morphogenetic protein 2/Smad, phosphoinositide 3-kinase/Akt and protein kinase A, play a role in this. Furthermore, these agents modulate inflammation, reduce apoptosis, and improve stem cell functions even under diabetic or inflammatory conditions. Exendin-4 facilitated tenogenic and insulin-producing cell differentiation, particularly in engineered scaffolds or genetically engineered human mesenchymal stem cells.

**Conclusion:**

Glucagon-like peptide-1 receptor agonists modulate key pathways in human mesenchymal stem cells to influence survival, differentiation, and metabolic function, suggesting promising therapeutic potential beyond glycemic control. However, heterogeneous experimental designs and limited translational data necessitate further standardized and in vivo research to define clinical applications.

##  Introduction

Glucagon-like peptide-1 (GLP-1) is a gut-derived hormone that plays a crucial role in glucose metabolism and insulin secretion. It is released in response to food intake and functions by stimulating insulin release in a glucose-dependent manner, inhibiting glucagon secretion, and slowing gastric emptying, thus contributing to the regulation of blood glucose levels [1]. Due to its effects on glucose metabolism, GLP-1 and its synthetic agonists have become key pharmacological agents in the treatment of type 2 diabetes (T2D) [2].

Over recent years, GLP-1-receptor-agonists (GLP-1-RA) have gained significant attention not only for their antidiabetic properties but also for their weight reducing effects [3].

GLP-1-RA such as liraglutide [4], semaglutide [5], and the dual agonist tirzepatide [6], activating both the GLP1- and the gastric-inhibitory polypeptide receptor (GIP-R), have been shown to significantly reduce body weight, making them a promising therapeutic option for the treatment of obesity.

**Fig. 1:**
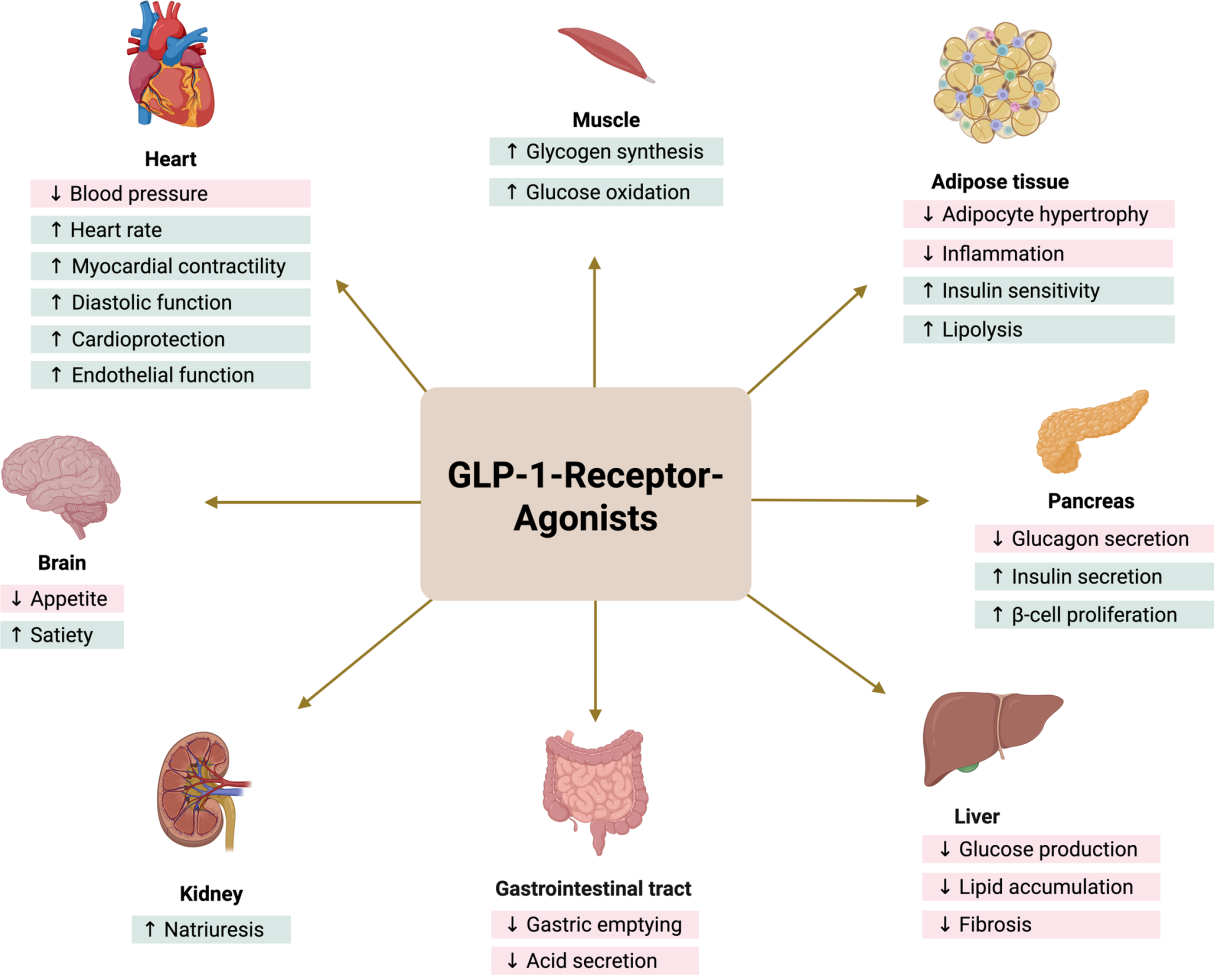
Effect of GLP-1-RA on target tissues (created on Biorender)

While the metabolic effects of GLP-1-RA, particularly on weight loss and glucose regulation, are well-characterized (Fig. 1), their potential influence on stem cells biology, especially human mesenchymal stem cells (hMSC) are not yet fully understood. hMSC, known for their regenerative and differentiation capabilities, represent a promising cell type for tissue repair and regeneration, particularly in adipose tissue, bone and cartilage [7].

Given the regenerative potential of hMSC, understanding how GLP-1-RA affect these cells is crucial for determining further implications of these drugs on tissue homeostasis and metabolic health.

This systematic review aims to summarize and critically evaluate the available in vitro studies investigating the effects of GLP-1-RA on hMSC. By analyzing the existing literature, we seek to elucidate the underlying mechanisms through which GLP-1-RA modulate hMSC function and to assess the potential implications of these effects for regenerative medicine.

## Methods

This systematic review was conducted according to the PRISMA (Preferred Reporting Items for Systematic Reviews and Meta-Analyses) guidelines [8].

### PICO Framework

Population: in vitro human mesenchymal stem cells (hMSC).

Intervention: Treatment with GLP-1-RA.

Control: Control Group (e.g. standard cell culture medium).

Outcome: Effects of GLP-1-RA on cell properties.

### Search Strategy

A comprehensive literature search was conducted using the National Library of Medicine database (www.pubmed.ncbi.nlm.nih.gov*)* without applying any language or publication date restrictions. Specifically, we used a combination of keywords such as “Glucagon-like-peptide-1-receptor agonists”, “mesenchymal stem cells” alongside different spellings and variations as well as relevant Boolean operators (AND/OR) to identify pertinent studies.

Search string:

(Glucagon-Like Peptide-1 Receptor Agonist* OR GLP-1-RA* OR GLP-1-R* OR GLP-1 Receptor Agonist* OR Liraglutide OR Semaglutide OR Tirzepatide OR Exenatide OR Exendin-4) AND (“Stem Cells“[Mesh] OR stem cell* OR stromal cell* OR adult progenitor cell* OR hASC OR hASCs OR ASC OR ASCs OR adipose-derived stem cell* OR ADSC* OR MSC OR mesenchymal stem cell* OR mesenchymal stromal cell* OR mesenchymal progenitor cell* OR bone-marrow derived mesenchymal stem cell* OR BD-MSC* OR umbilical cord tissue derived mesenchymal stem cell* OR UC-MSC*)

The date of the search was June 30th, 2025.

### Eligibility Criteria

Inclusion criteria:

- Full-text original articles.
- Focus on the influence of GLP-1-RA (e.g., Exenatide, Semaglutide, Liraglutide) on hMSC.
- In-vitro studies.
- English language.

Exclusion criteria:

- Clinical trials.
- In vivo studies.
- Reviews.
- Letters and comments.
- Case reports.
- Paper not available as full text.
- Retraction.
- Not peer-reviewed.
- Usage of animal-derived stem cells or cell lines.

### Screening and Article Selection

Two independent researchers screened titles and abstracts of all retrieved studies based on the eligibility criteria. Full-text articles of potentially eligible studies were assessed for final inclusion. Disagreements between the researchers were resolved, also with the involvement of a third and fourth researcher.

Data extraction was performed independently by two researchers using a predesigned data extraction form. The extracted information included:

- Study characteristics (authors, title, citation ID, abstract).
- GLP-1-RA details (name, concentration).
- hMSC characteristics.
- Experimental outcomes (proliferation, differentiation, migration, signaling cascades, tissue engineering).
- Key findings.

### Quality Assessment

The quality of the included studies was evaluated using an adapted Quality Assessment Tool For In Vitro Studies (QUIN) risk-of-bias tool for in vitro studies, originally designed for dentistry studies [9]. This tool evaluates potential sources of bias across twelve domains:

- Domain 1: Clearly stated aims/objectives.
- Domain 2: Detailed explanation of sample size calculation.
- Domain 3: Detailed explanation of sample handling.
- Domain 4: Details of comparison group.
- Domain 5: Detailed explanation of methodology.
- Domain 6: Operator details.
- Domain 7: Randomization.
- Domain 8: Method of measurement of outcome.
- Domain 9: Outcome assessor details.
- Domain 10: Blinding.
- Domain 11: Statistical analysis.
- Domain 12: Presentation of results.

Criteria such as study design, reporting of cell culture conditions, and reproducibility of findings were assessed by two reviewers independently. Judgements for risk of bias in each domain were categorized as low, moderate or high. Disagreements were resolved through discussion.

Studies were not excluded based on their risk of bias rating, but results from those with higher risk should be interpreted with caution.

## Results

### Search Results

A comprehensive literature search identified a total of 379 articles. These articles were initially screened based on their titles and abstracts. During this stage, 300 articles were excluded due to irrelevance to the research question or failure to meet the inclusion criteria (22 clinical trials, 43 reviews or other unfitting literature, 112 wrong cell type, 49 wrong focus, 10 wrong drugs, 57 in vivo, 2 retractions, 3 no abstract and 2 not peer reviewed yet).

Of the initial 79 articles identified, 3 were excluded because the full texts were unavailable.

**Fig. 2:**
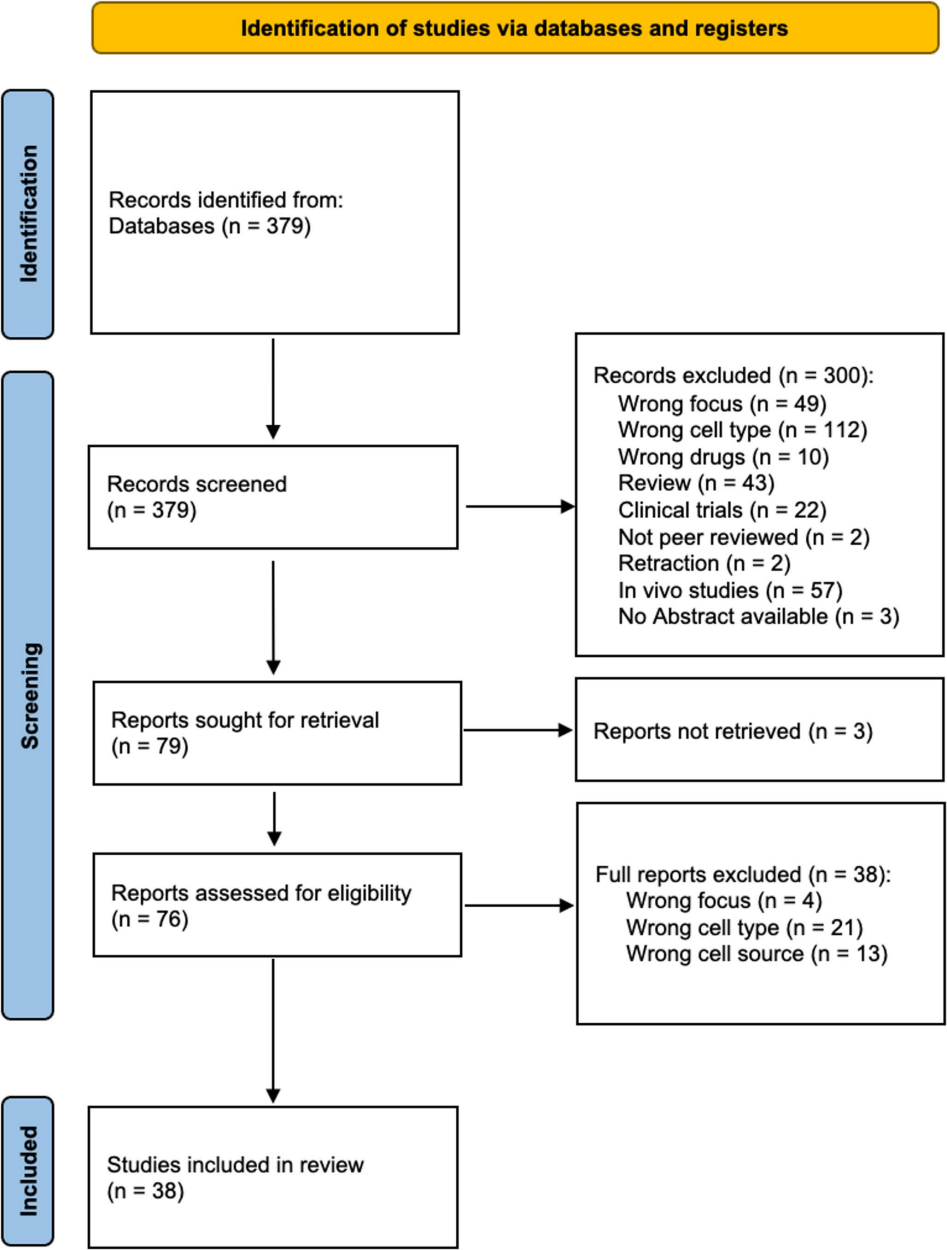
PRISMA 2020 flowchart

The remaining 76 articles underwent a full-text screening process, during which 38 articles were excluded for the following reasons: 4 wrong focus, 13 cells of animal origin and 21 due to an unfitting cell type. Thus, a total of 38 articles met the inclusion criteria and were included in the final analysis (Fig. 2).

### Quality Assessment

**Fig. 3:**
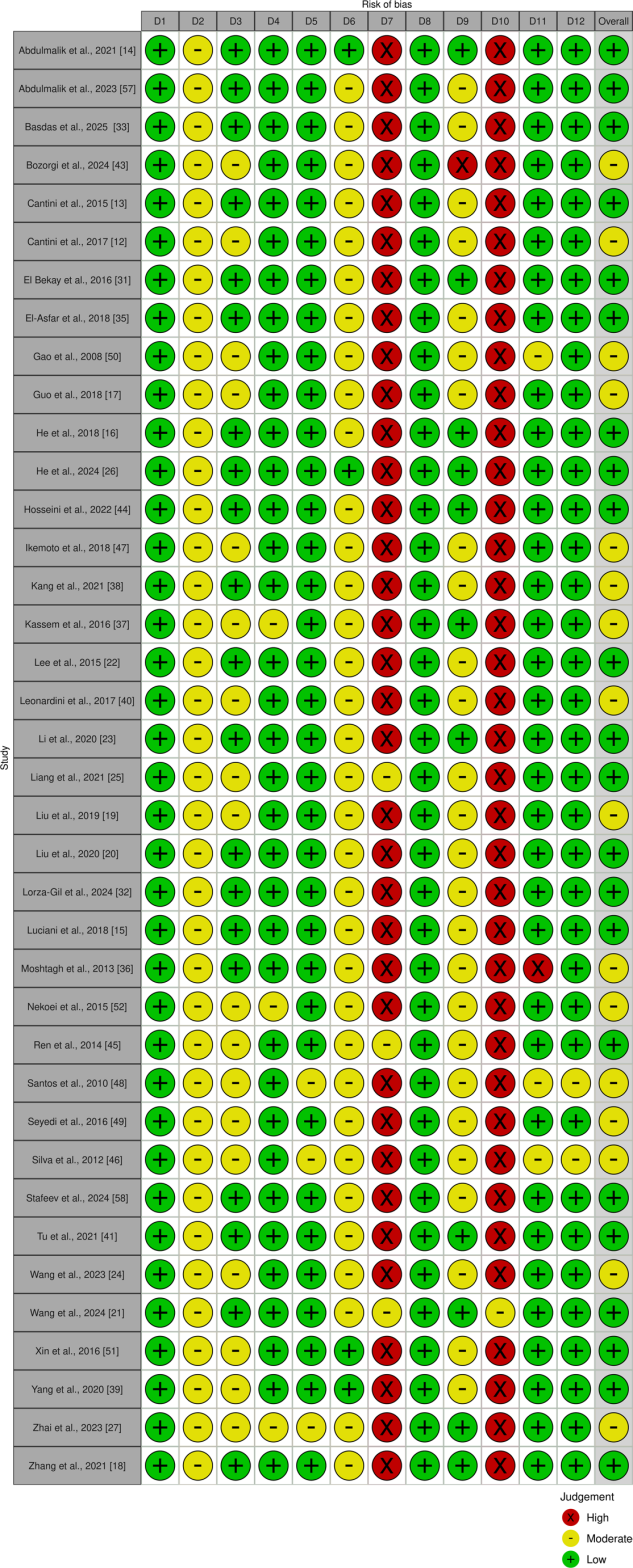
Quality assessment of included studies in the systematic review, created using the robvis tool (D1: Clearly stated aims/objectives (domain 1), D2: Detailed explanation of sample size calculation (domain 2), D3: Detailed explanation of sample handling (domain 3), D4: Details of comparison group (domain 4), D5: Detailed explanation of methodology (domain 5), D6: Operator details (domain 6), D7: Randomization (domain 7), D8: Method of measurement of outcome (domain 8), D9: Outcome assessor details (domain 9), D10: Blinding (domain 10), D11: Statistical analysis (domain 11), D12: Presentation of results (domain 12))

The results of the quality assessment were visualized using R and the robvis tool (Fig. 3), which provides a graphical representation of the risk of bias across studies and criteria [10]. Most studies were judged to have a low risk of bias, but a few studies however showed moderate risk of bias.

Notably, domain 7 and 10 assessing randomization and blinding were frequently rated as “high risk of bias”. This likely reflects methodological characteristics inherent to in vitro research, where randomization and blinding are usually not applicable, rather than a true source of systematic bias. Thus, this ratings should be interpreted with caution in the context of in vitro study design.

## Discussion

### Proliferation and Migration

HMSC exhibit remarkable capabilities of proliferation and self-renewal. Equally important is their capacity for migration, which is essential for facilitating wound healing, tissue regeneration, and repair following activation through tissue-mediated signaling [11]. These properties can be affected by pharmacological agents or growth factors, such as different GLP-1-RA in various concentrations.

#### GLP-1

Cantini et al. investigated the effects of GLP-1(9-36), a metabolite produced by N- terminal dipeptide cleavage of native GLP-1(7-36), which exhibits very low affinity for the GLP-1-receptor, in comparison to GLP-1(7-36) and liraglutide. GLP-1(9-36) and GLP-1(7-36) in the concentrations of 1, 10 and 100 nM showed statistically significant inhibition of cell growth in human adipose tissue derived stem cells (hASCs), without any dose-dependent effects. Interestingly, inhibitory effects of GLP-1(7-36), but not of GLP-1(9-36), on cell counts were reverted by the GLP-1-R-Antagonist Exendin (9-36) [12].

Regarding glucose-metabolism, two studies found that GLP-1(9-36), GLP-1 (7-36) and Liraglutide significantly inhibited insulin-stimulated glucose uptake [12, 13], with Liraglutide showing a dose dependent inhibition, with a maximum at a concentration of 1000 nM [12, 13].

#### Exendin-4

HMSC treatment with GLP-1-RA Exendin-4 (Ex-4), a 39 amino acid peptide originally isolated from Heloderma suspectum venom [14], did not have an influence on GLP-1 receptor nor hMSC surface receptor expression used for cell characterization [14, 15]. Nevertheless, some studies suggest that Ex-4 significantly stimulates hMSC proliferation in a dose- and time-dependent-manner, particularly at concentrations between 10 nM and 100 nM, as shown in cell viability assays 3-(4,5-dimethylthiazol-2-yl)−5-(3-carboxymethoxyphenyl)−2-(4-sulfophenyl)−2 H-tetrazolium (MTS) and Cell Counting Kit-8 (CCK-8), as well as DNA analysis 5-ethynyl-2′-deoxyuridin (EdU) incorporation and PicoGreen assay [14–16]. When exposed to Ex-4, cell cycle analysis revealed an increase of cells in S (synthesis)-Phase [15, 16] and proliferation related markers (*cyclin D3*, cyclin-dependent kinase 4 (*CDK4*), *c-Myc* and *c-fos*) increased, while cell cycle inhibitors (*p21Cip1* and *p27Kip1*) were downregulated [16]. Flow cytometry also demonstrated greater SRY-box transcription factor 2 (*SOX2*) expression, a gene involved in regulation of cell proliferation, in hMSC when treated with Ex-4 [15]. Under high glucose conditions Ex-4 was able to reverse glucose-induced suppression of proliferation in human Periodontal ligament stem cells (PDLSC) [17] and reduced cellular senescence in hMSC as confirmed by EdU staining, flow cytometry and cell cycle analysis [18]. However, two exceptions were reported where Ex-4 10 nM had no effect on PDLSC proliferation under Lipopolysaccharides (LPS) induced inflammatory conditions [19] and one study showed short-term exposure with Ex-4 100 nM for 24–72 h had no effect on hMSC proliferation [15].

#### Liraglutide

In contrast to the proliferative effects observed with Exendin-4, Liraglutide predominantly exhibited inhibitory effects on hMSC proliferation [12, 13, 20]. Especially at concentrations of 10–100 nM Liraglutide lead to a significant reduction in proliferation and DNA synthesis as observed in cell counting, MTS, 3-(4,5-di methylthiazol-2-yl) −2,5-diphenyltetrazolium bromide (MTT) and [³H]-thymidine incorporation assays [13, 20]. When adding GLP-1-R-Antagonist Exendin (9-36), the effects of Liraglutide were reversed, indicating receptor-mediated action [13]. Exceptions were observed when Liraglutide at 10 nM enhanced the proliferation of hMSC pretreated with LPS to mimic an inflammatory setting [4], and in experiments performed by Wang et al., where Liraglutide at 10 nM showed no effect on the proliferation of human bone marrow-derived stem cells (hBMSC) [21]. Interestingly, the same group could demonstrate that treatment with Liraglutide at 10nM enhanced the migration capacity of hBMSC, suggesting context-specific effects of the drug beyond its influence on proliferation [21].

These findings highlight that GLP-1-RA such as Ex-4 can promote hMSC proliferation, while native GLP-1 forms and Liraglutide predominantly exert inhibitory effects on proliferation and glucose uptake, with varying results under different conditions, highlighting their context-dependent role in hMSC behavior and metabolic regulation.

### Differentiation

GLP-1 and its analogues have been shown to modulate adipogenic and osteogenic differentiation in various hMSC populations, influencing both lipid metabolism and bone formation through distinct signaling pathways. In addition to these established differentiation trajectories their role in directing hMSC differentiation toward insulin-producing cells (IPC) will also be discussed in a later section.

#### Osteogenesis

During osteogenic differentiation native GLP-1 100 nM increased alkaline phosphatase (ALP) activity, mRNA expression and calcium deposition in human adipose derived stem cells (hASC) [22]. Under high glucose conditions, GLP-1 at 100 nM enhanced the expression of osteogenic markers (collagen type I (*COL1*), osteocalcin (*OCN*), runt-related transcription factor 2 (*RUNX2*) and activated the Wingless-type MMTV integration site family member (*Wnt*)/ß-catenin pathway, known to play a key role in osteogenesis, as shown by Li et al. [23].

Ex-4 at 10 nM promoted mineralization and upregulated osteogenic markers *RUNX2*, *ALP* and osterix (*OSX*) in PDLSCs. Furthermore, it reversed osteogenic inhibition induced by high glucose or inflammatory conditions. These effects are suggested to occur through the activation of mitogen-activated protein kinase (MAPK) and Wnt/ß-catenin signaling pathways [17, 20, 24, 25]. Furthermore, Luciani et al. demonstrated that the timing of Ex-4 administration critically influences osteogenic differentiation of hMSC. While co-treatment with 300 nM Ex-4 during differentiation suppressed osteogenic marker expression of *RUNX2*, bone morphogenetic protein 2 (*BMP2*), *ALP* and matrix deposition, early exposure prior to differentiation start or at the time of cell isolation, enhanced osteogenic marker expression [15]. A completely different approach was employed by He et al. [26], who engineered hMSC to overexpress Ex-4 (hMSC-E4) using lentiviral transduction. HMSC-E4 retained typical hMSC morphology, surface markers, and viability, but showed a 10,000-fold increase in *Exendin-4* mRNA and a 20-fold rise in protein levels. This modification resulted in enhanced ALP-activity, mineralization, and osteogenic marker expression (*ALP*,* COL1A1*,* RUNX2*) via autocrine signaling. Furthermore, they explored the role of hMSC-E4 in endochondral osteogenesis using an in vitro model embedding hMSC in a COL1 sponge matrix. HMSC-E4 promoted enhanced cartilage formation, as evidenced by intensified Safranin-O and type II collagen (COL2) staining, higher Bern scores, and increased expression of type X collagen (*COL10A1*) and *COL1A1*. Micro-Computer tomography confirmed greater bone volume, highlighting improved osteogenesis compared to control hMSC. Single-cell RNA sequencing further revealed a shift toward osteogenic lineages, cartilage progenitor cells and pre-osteoblasts, underscoring hMSC-E4’s capacity to accelerate chondrogenic maturation and endochondral bone formation [26].

Liraglutide at 10 nM and 100 nM also promoted osteogenic differentiation in hBMSC and Human dental pulp stem cells (hDPSC) through enhancing ALP activity, mineralization and osteogenic marker expression [21, 27]. Experiments indicate that it acts through the BMP2/Smad/RUNX2 pathway [21] and long intergenic non-coding RNA 968 (LINC00968)/micro-RNA-3658 (miR-3658)/RUNX2 pathway [27].

Rodent MSC also exhibit ameliorated osteogenic differentiation when treated with GLP-1-RA, mirroring findings in human MSC. Zhou et al. [28] applied 100nM Ex-4to rat STRO-1-positive BMSC, which led to superior mineralization and osteoblast maturity. In another study treatment with 20nM Ex-4 on rat adipose-derived MSC additionally elevated OPG and RUNX2 protein levels, while reducing osteoclast activity [29].

Similarly, 100nM Semaglutide promoted osteogenesis in rat BMSC via WNT-beta-Catenin signaling, which resulted in promoted mineral deposition and *RUNX2/OCN* gene expression [30]. Together these studies suggest that GLP-1-RA exert comparable results with little evidence for species-specific differences.

#### Adipogenesis

In contrast to their promotive effects on osteogenesis, GLP-1 and its analogues GLP-1(9-36), GLP-1(7-36) and Liraglutide demonstrated a dose-dependent inhibition of adipogenic differentiation in hASCs. The most pronounced inhibitory effects were observed at concentrations of 10 and 100 nM for each compound. This inhibition was evidenced by Oil Red O and AdipoRed staining, and by the downregulation of key adipogenic markers, including peroxisome proliferator-activated receptor gamma (*PPARγ*), lipoprotein lipase (*LPL*), fatty acid-binding protein 4 (*FABP4*), and CCAAT/enhancer-binding protein alpha (*C/EBPα*) [12, 13, 22, 23].

Interestingly, Liraglutide 10−100nM increased adiponectin (*ADIPOQ)* expression [13] and in one study it led to an initial upregulation of *PPARγ* and *C/EBPα* followed by a significant downregulation, while preadipocyte marker Pref-1 was gradually increased [20]. Anti-adipogenic effects were also seen under high glucose conditions [23]. Experiments indicate that different pathways like extracellular signal-regulated kinase (ERK) and WNT [20, 23] signaling pathways play a role in this. Furthermore, Liraglutide treatment showed no change in triglyceride levels, but it led to an increased glycerol release over time, indicating enhanced lipolysis [20]. Supporting these findings Bekay et al. demonstrated that native GLP-1 10nM downregulated adipogenic markers (*PPARγ*, adipose differentiation-related protein (*ADRP*), *FABP4*) and lipogenic genes (*LPL*, fatty acid synthase (*FASN*), sterol regulatory element-binding protein 1 (*SREBP1*), while increasing lipolytic markers hormone-sensitive lipase (*HSL*), Perilipin and adipose triglyceride lipase (*ATGL*) in Stromal Vascular Fraction cells (SVFCs) of adipose tissue during adipogenic differentiation [31]. The same group showed that GLP-1 continued to suppress lipogenesis and increase lipolysis in day 14 differentiated cells from subcutaneous adipose tissue (SAT) and visceral adipose tissue (VAT), with the effects being more pronounced in SAT [31]. When adding GLP-1-R-Antagonist Exendin(9-36), Cantini et al. [13] showed that Liraglutide mediated effects were suppressed.

Unlike the consistent inhibition seen with GLP-1 analogues, Ex-4 has shown variable effects on adipogenic differentiation. As previously noted for its osteogenic effects, Luciani et al. [15] demonstrated that the timing of Ex-4 exposure is a critical determinant of its influence on adipogenic differentiation of hMSC. In this respect, 300 nM Ex-4 during differentiation reduced lipid accumulation and downregulated the adipogenic markers *PPARγ*, *FABP4*, whereas early exposure at cell isolation or prior to induction increased these markers, indicating pro-adipogenic priming effects. In differentiated hMSC, Ex-4 enhanced lipolysis by increasing the expression of the lipolytic marker *ATGL* [15]. Also 10–100 nM Ex-4 treatment was reported to increase lipid droplet number while reducing their size. This was accompanied by the induction of both white and brown adipocyte markers, alongside the downregulation of anti-adipogenic genes like delta-like 1 homolog (*DLK1*), forkhead box protein O1 (FOXO1), and Wnt family member 3 A (*WNT3A*) in hMSC [16].

Lorza-Gil et al. [32] studied the effects of Tirzepatide, a dual gastric inhibitory polypeptide-receptor agonist (GIP-RA) and GLP-1-RA, and gastric inhibitory polypeptide (GIP), on pancreatic adipose-tissue derived SVF cells in 2D and 3D culture. GIP at 20 nM had no major effect on adipogenic gene expression, while 10 nM Tirzepatide reduced insulin receptor (INSR) and *ADIPOQ* levels. Furthermore, both GIP 100 nM and Tirzepatide at 10 nM stimulated lipolysis through cyclic adenosine monophosphate (cAMP)/protein kinase A (PKA) pathway, as evidenced by increased glycerol release and HSL phosphorylation on day 19 differentiated cells. Tirzepatide and GIP treatment lead to significantly lower interleukin-1 beta (IL1B) levels and Tirzepatide further lowered interleukin-6 (IL-6) and monocyte-chemoattractant protein-1 (MCP-1) levels, highlighting distinct tissue-specific regulatory roles in pancreatic adipose tissue metabolism and inflammation [32].

In the study from Basdas et al. [33], the effect of another GLP-1-RA, Semaglutide at 1 nM, on adipogenic differentiation of stromal vascular cells isolated from SAT and epicardial adipose tissue (EAT) was examined. In EAT the drug reduced *FABP4* and perilipin 1 (*PLIN1*), but not *ADIPOQ* expression and on SAT it reduced *PPARγ* coactivator 1-alpha (*PGC1α)* expression. Additionally, it lowered inflammation markers and fibrosis related genes, like IL6, secretory phospholipase A₂ and matrix-metalloproteinase 9. Furthermore, it slightly reduced the increase of glucose transporter types 1 and 4 (GLUT1/4) during adipogenesis and led to a rise of GLP-1 mRNA, though protein levels remained unchanged. In EAT, it altered protein profile, increasing protective proteins like caveolin-1 (CAV1) and voltage-dependent anion-selective channel protein 3 (VDAC3), while reducing proinflammatory ones. These findings highlight Semaglutide’s potential to modulate adipogenesis and exert anti-inflammatory and protective effects, especially in inflamed EAT.

While in hMSCs predominantly anti-adipogenic effects were observed, Wang et al. [34] demonstrated that murine MSCs treated with liraglutide (10–100 nM) during brown adipogenic differentiation showed enhanced lipid accumulation and upregulation of adipogenic markers. This indicates that differences between species may influence outcomes, despite using the same concentration range.

In summary, GLP-1 and its analogues have been shown to promote osteogenic differentiation, suppressing adipogenesis and act anti-inflammatory across multiple stem cell sources. Data suggests that these effects are being mediated through key pathways such as WNT/ß-catenin, MAPK, BMP/Smad and ERK. It is important to highlight that their effects on differentiation are often context- and timing-dependent and may vary on hMSC derived from different sources (Tables 1 and 2). This highlights the complexity of GLP-1-strategies in regenerative medicine and metabolic modulation.

**Table 1:** Comparison of effects of GLP-1-RA on osteogenesis

Source	Cell source	GLP-1-RA and concentration used	Differentiation protocol outline	Duration (days)	Additional information
Lee et al. [22]	hASC	native GLP-1 (10nM and 100nM)	Growth medium supplemented with 100 nM dexamethasone, 50 µM ascorbate acid, and 10 mM β-glycerophosphate sodium	21	GLP-1 100nM increased ALP mRNA (day 7), ALP activity (day 10) and calcium deposition (day 14)
Li et al. [23]	hASC	GLP-1 (7–36) (0.1,1,10 and 100 nM)	osteogenesis: DMEM/F12 supplemented with 5 mM β-glycerol phosphate disodium salt, 100 nM dexamethasone and 50 µM L-ascorbic acid under high glucose conditions (25 mM) to mimic hyperglycemic conditions in vitro	28	GLP-1 100 nM promotes osteogenesis via Wnt/β-catenin, increasing osteogenic markers (RUNX2, OCN, COL1, beta-catenin) and downregulated GSK-3β protein
Guo et al. [17]	PDLSC	Ex-4 (10 nM)	α-Minimum Essential Medium (αMEM) containing 5%, FBS, 50 µg/mL ascorbic acid, 1 µM dexamethasone, and 3 mM β-glycerophosphate conditions (normoglycemic, normoglycemic plus 10nM Ex-4, hyperglycemic and hyperglycemic plus 10nM Ex-4)	21	Ex-4 10 nM increased mineralization in Alizarin Red staining and osteogenic genes (RUNX2, ALP, OSX); also reversed osteogenic inhibition under HG conditions
Wang et al. [21]	PDLSC	Ex-4 (10 nM)	α-MEM, 10% FBS, 1% streptomycin solution, 1 µM dexamethasone, 50 µg/mL ascorbic acid, and 3 µM β-glycerophosphate under normoglycemia (5.5 mM D-glucose) and hyperglycemia (30 mM)	21	Ex-4 10 nM increased osteogenic genes (ALP, RUNX2, and OSX); reversed inhibitory effect of HG conditions; also seen in ALP activity (day 7 and 21). upregulated the activity of MAPK and Wnt signaling pathways
Liang et al. [25]	PDLSC	Ex-4 (10 nM)	basic media with 10–8 mol/L dexamethasone, 50 mg/L ascorbic acid and 10 mmol/L β-glycerophosphate Osteogenic induction medium with Stromal cell-derived factor-1(SDF- 1), Ex-4, or SDF-1 + Ex-4	21	Ex-4 showed enhanced mineralization in Alizarin Red S staining and increased ALP activity; increased Runx2, OCN, OSX, Col1 expression. synergistic with SDF-1
Luciani et al. [15]	hMSC	Ex-4 (100 and 300 nM)	DMEM with 2% FBS, 100 µM ascorbic acid, 10 mM β-glycerophosphate and 10 nM dexamethasone **Experiment 1**: exposed to Ex-4 during differentiation **Experiment 2**: cells were pre-treated for 7 days with Ex-4, then induced to specific differentiation **Experiment 3**: long term pretreatment with 100 or 300 nM Ex-4 at the time of cell isolation	21	**Experiment 1**: Ex-4 300 nM decreased osteogenic markers (RUNX2, ALP and BMP2) and showed less mineralization in Alizarin Red **Experiment 2**: pretreatment prevented downregulation of osteogenic markers **Experiment 3**: increase of osteogenic markers (RUNX, BMP2, ALP) compared to hMSC isolated in standard medium
He et al. [26]	Human umbilical cord MSC	hMSC modified to overexpress Ex-4	**osteogenesis**: 500 µM ascorbic acid, 10 mM β-glycerophosphate, and 100 nM dexamethasone **endochondral osteogenesis**: hMSC were embedded in a COL1 sponge matrix; *chondro-inductive medium*: 10 ng/ml TGF-β3, 100 nM dexamethasone, and 10 µM ascorbic acid; *hyperchondro-inductive medium*: 50 µM L-thyroxin, 100 nM dexamethasone, 50 pg/ml interleukin 1-β and 0.01 M β-glycerophosphate	**osteogenesis**: 21 **endochondral osteogenesis**: 42	**osteogenesis**: autocrine signaling resulted in enhanced ALP-activity, mineralization, and osteogenic marker expression (ALP, COL1A1, RUNX2) **endochondral**: hMSC-E4 showed intensified Safranin-O and type II collagen (COL2) staining, higher Bern scores, increased expression of type X collagen (COL10A1) and COL1A1, and greater bone volume
Wang et al. [21]	hBMSC from diabetic and non-diabetic patients	Liraglutide (10, 100, 1000, 10000 nM)	α-MEM supplemented with 100 µM L-ascorbic acid, 2 mM β- glycerophosphate, 10% fetal calf serum, NY, and 1% dual antibiotics	21	Liraglutide 10 nM showed higher ALP activity (3, 5, 7 days) and stronger ALP staining (10 days); higher doses showed inhibitory effects Liraglutide 10 and 100 nM showed more intense Alizarin red staining (21 days) Liraglutide 10 nM increased osteogenic markers (ALP, COL1, and OCN) Liraglutide 10 nM activates BMP2/Smad/Runx2 pathway
Zhai et al. [27]	hDPSC	Liraglutide (25, 50, 100 and 200 nM)	10 − 4 M L-ascorbic acid, 10 mM β-glycerophosphate and 10 − 8 M dexamethasone	7	Liraglutide 100 nM and 200 nM stimulated ALP activity, more prominent at 100 nM Liraglutide 100 nM increased calcium deposition in alizarin red staining and increased osteogenic markers (RUNX22, COL1l, osteonectin, OCN) as well as RUNX2 protein levels Liraglutide 100 nM stimulates RUNX2 expression by activating LINC00968/miR-3658 pathway

**Table 2:** Comparison of effects of GLP-1-RA on adipogenesis

Source	Cell source	GLP-1-RA ´ and concentration used	Differentiation protocol outline	Duration (days)	Additional information
Lee et al. [22]	hASC	native GLP-1 (10nM and 100nM)	Growth medium supplemented with 1 µM dexamethasone, 1 mM 3-isobutyl-1-methylxanthine, 10 ng/ mL insulin and 60 µM indomethacin	14	GLP-1 100nM decreased lipid accumulation (day 14) and downregulated expression of adipocyte protein 2, PPAR-γ and LPL (after day 3 and 7)
Li et al. [23]	hASC	GLP-1 (7–36) (0.1,1,10 and 100 nM)	DMEM/F12 supplemented with 0.5 mM 3-isobutyl-1- methylxanthine for the first 3 days (IBMX),1 µM dexamethasone, 5 µg/ml insulin, 0.2 nM triiodothyronine, 33 µM biotin, 17 µM calcium pantothenate, and 10 mg/L human transferrin under high glucose conditions (25 mM) to mimic hyperglycemic conditions in vitro	21	GLP-1 100 nM inhibited adipogenesis, decreasing lipid droplets Oil Red O staining and decreasing PPAR-γ, C/EBP-α, GSK-3β
Liu et al. [20]	hASC	Liraglutide (0.1, 1, 10, 100 nM)	DMEM/F12 (1:1), 1µmol/L dexamethasone, 1µmol/L insulin, 10 µg/mL transferrin, and 0.1mmol/L IBMX 3 days then transferred to maintenance medium, same as differentiation medium without IBMX	21	Liraglutide 100 nM: decreased lipid droplet number, PPAR-γ and C/EBPα mRNA: day 7 upregulation, day 21 downregulation, PPAR-γ, C/EBPα and preadipocyte factor 1 protein levels increased Liraglutide 100 nM downregulates mRNA levels of GSK-3α and GSK-3β levels during differentiation phosphorylated GSK-3β in liraglutide group was upregulated Glycerol and TAG assay Liraglutide 0.1, 1, 10, 100nM: slight increase of triglyceride content, but no statistical significance; glycerol content gradually increased
Luciani et al. [15]	hMSC	Ex-4 (100 and 300 nM)	DMEM with 10% FBS, 1µM dexamethasone, 0.5 mM methyl-isobutylxanthine and 10 µg/mL insulin, after 48–72 h replaced with an adipogenic maintaining medium containing 10 µg/mL insulin for 24 h **Experiment 1**: exposed to Ex-4 during differentiation **Experiment 2**: cells were pre-treated for 7 days with Ex-4, then induced to specific differentiation **Experiment 3**: long term pretreatment with 100 or 300 nM Ex-4 at the time of cell isolation	3–4	**Adipogenesis** Experiment 1: Ex-4 300 nM reduced adipogenic markers (PPARγ and FABP4) and decreased lipid droplets in Oil Red O Experiment 2: pretreatment prevented downregulation of adipogenic markers **Experiment 3**: increase of adipogenic markers (FABP4, PPARγ) compared to hMSC isolated in standard medium
Cantini et al. [12]	hASC	GLP-1(9–36), GLP-1(7–36), Liraglutide 10 nM	0% FBS-DMEM, 0.5 mM IBMX, 1 mM dexamethasone, 200 mM indomethacin and 10 mM insulin for 2 weeks, then 10% FBS- DMEM containing 1.7 mM insulin for one week	21	GLP-1(9–36), GLP-1(7–36) reduced lipid droplets in Oil Red O and AdipoRed staining GLP-1(9–36), GLP-1(7–36), Liraglutide reduction in FABP4 and increased adiponectin expression only GLP-1(7–36) effect was reverted GLP-1-R-Antagonist exendin (9–39)
Cantini et al. [13]	hASC	Liraglutide (1, 10 and 100 nM)	adipogenesis: 10% FBS–DMEM, 0.5 mM IBMX, 1 mM dexamethasone, 200 mM indomethacin and 10 mM insulin for 2 weeks, then shifted to 10% FBS–DMEM containing 1.7 mM insulin for another week	21	Liraglutide 10 and 100 nM reduced differentiated adipocytes in Oil Red O and AdipoRed Liraglutide reduced FABP4 but increased APOM GLP-1-R-Antagonist Exendin 9–39 reverted effects of Liraglutide
El Bekay et al. [31]	SVF-cells from VAT and SAT	GLP-1 (10, 100 and 1000 nM)	expansion medium supplemented with 0.5 mM IBMX, 1.0 µM dexamethasone, 10 µM insulin and 200 µM Indomethacin SVF-cells were induced to adipogenic differentiation for 15 days with or without 10 nM GLP-1 Also, day 14 differentiated adipocytes were exposed to different doses of GLP-1 (10, 100 and 1000 nM)	15	GLP-1 10 nM decreased adipogenic markers ADRP and FABP4, while increasing HSL and perilipin mRNA during adipogenesis, effects were observed starting at day 6 Day 14 in vitro differentiated cells: SVF-cells from SAT decrease of adipogenic markers (PPARγ, FABP4), lipogenesis markers (LPL, FASN) and lipolysis marker (ATGL) SVF-cells from VAT GLP-1 100 and 1000 nM decreased adiponectin, while increasing AZGP1 mRNA
Lorza-Gil et al. [32]	Human pancreatic adipose tissue-derived SVF	Tirzepatide (10 nM)	7 days in induction media consisting of DMEM/Ham’s nutrient mixture (1:1) supplemented with (mmol/l): 17 mmol/l pantothenate, 1mmol/l biotin, 0.025 mmol/l apotransferrin, 1 mmol/l insulin, 500 mmol/l IBMX, 1 mmol/dexamethasone, 5 mmol/l troglitazone, 50 mmol/indomethacin; 5% FBS and 1% penicillin/streptomycin on day 12 in differentiation media consisting of induction medium deprived of IBMX, dexamethasone and indomethacin	19	Starting at day 7 of differentiation organoids were supplemented with GIP (20 nM) and Tirzepatide (10 nM): Tirzepatide reduced the mRNA levels of INSR and ADIPOQ On day 19 differentiated organoids and 2D monolayer culture GIP (100 nM) and tirzepatide (10 nM) for 3 h both increased lipolysis (increase in glycerol release and phosphorylation of HSL) On day 19 differentiated organoids GIP (100 nM) and tirzepatide (10 nM) for 24 h ->GIP reduced adiponectin, Tirzepatide reduces IL-6 and MCP-1 levels, GIP and Tirzepatide decreased IL1B
Basdas et al. [33]	stromal vascular cells isolated from SAT and epicardial adipose tissue (EAT)	Semaglutide (1 nM)	Medium 199 with Earle’s Salts (0.25 mL, 10% fetal bovine serum, insulin (5 ug/mL), dexamethasone (250 nmol/L), methylisobutylxanthine (0.5 mmol/L), and thiazolidinedione (1 mmol/L)	15	**EAT** Semaglutide 1 nM downregulated adipocyte markers (FABP4, (PLIN1), ADIPOQ levels were maintained, Inhibited increase of proinflammatory marker Secretory Phospholipase A₂(sPLA2), Reduced GLUT1 and GLUT4 mRNA expression, Upregulated GLP1R mRNA but not protein levels **SAT** Semaglutide 1 nM downregulated PGC1a, inflammation-related genes (IL6, sPLA2) and fibrosis-related gene MMP9 Reduced GLUT1 and GLUT4 mRNA expression Upregulated GLP1R mRNA but not protein levels

### Signaling Cascade

As already elucidated in the previous section, GLP-1-RA have demonstrated significant effects on hMSC and other progenitor cells behavior, by modulating differentiation, enhancing cell survival and regulating inflammation through multiple intracellular pathways.

#### GLP-1

Native GLP-1 reduces adipogenesis via ERK-inhibition, downregulating *PPARγ*, *LPL*, *C/EBPα* and *FABP4* [12, 22, 23, 31]. Osteogenesis however is activated and shows increased expression of *ALP*, *COL1*, *OCN* and *RUNX2*. These effects are likely mediated by Wnt/​​β-catenin signaling, which is activated via increased β-catenin, proven by Li et al. by silencing glycogen synthase kinase 3 beta (GSK-3β) and the Wnt-pathway-inhibitor Dickkopf-1 (DKK-1) [23].

#### Exendin-4

Ex-4 has been shown to enhance pancreatic beta-cell marker expression (pancreatic and duodenal homeobox 1 (*PDX-1*), NK2 homeobox 2 (*NKX2.2)*, ISL LIM homeobox 1 (I*SL-1*) and v-Maf musculoaponeurotic fibrosarcoma oncogene homolog A (*MAFA))* [35–37]. Especially in PDLSC, Ex-4 has also proven to activate MAPK and WNT-signaling [19, 21, 24] as well as Hedgehog/Gli1-1 and nuclear factor kappa B (NF-κB) signaling, thus promoting osteogenic differentiation [15, 17, 21, 24, 26, 38]. Treatment of hMSC with 300 nM Ex-4 for 7 days upregulated leptin and its receptor in both undifferentiated and osteogenically induced cells. Transcriptome analysis further revealed that these effects were amplified by co-treatment with Stromal cell-derived factor 1 (SDF-1) [38].

A 7-day treatment with 100 nmol/l Ex-4 promoted upregulation of pro-adipogenic genes associated with both white and brown adipose tissue [16]. In contrast, another study found that late stage of differentiation exposure at 300 nmol/l led to upregulation of lipolytic adipose triglyceride lipase (ATGL), aligning with the known lipolytic effects of GLP-1-RAs in human adipocytes. Additionally, when Ex-4 was administered during differentiation, it resulted in downregulation of *FABP4* and *PPARγ* [15].

At 10 nmol/l Ex-4 inhibits IκBα-phosphorylation and prevents NF-κB/p65 nuclear translocation, halving tumor necrosis factor alpha (TNF-α) and IL-6 both intracellularly and in the medium [19], thereby attenuating pro-inflammatory signaling and enhancing hMSC viability under inflammatory conditions. In a separate model, hMSC stimulation with 10 nM Liraglutide for 72 h led to increased levels of the anti-inflammatory cytokines interleukin-10 (IL-10) and tumor necrosis factor-stimulated gene 6 (*TSG-6*) in culture supernatant. Intracellularly, Liraglutide upregulated the expression of keratinocyte growth factor (*KGF*) in human chorionic membrane-derived mesenchymal stem cells, angiopoietin-1 (*ANG-1*) in human bone marrow-derived mesenchymal stem cells, and surfactant protein C (SPC) in human amniotic membrane-derived mesenchymal stem cells, highlighting distinct trophic responses dependent on hMSC tissue origin [39].

Regarding apoptosis, pretreatment with 20 nmol/l Ex-4 protects cardiac progenitor cells from palmitate induced cell death, and this protective effect extends even under hypoxic conditions but could be reversed by the GLP-1-R-Antagonist Exendin(9-36). Intracellular ceramide accumulation, a key indicator of apoptosis, was notably reduced. This can be linked to a significant reduction of serine palmitoyl transferase (SPT) protein levels and the downregulation of the ceramide-synthesis enzymes ceramide synthase 5 (*CERS5*) and delta(4)-desaturase as well as sphingolipid 1 (*DEGS1*) expression at both messenger-RNA and protein levels. These protective effects were reversed by small-interfering RNA targeting human GLP-1-R, the PKA inhibitor 14–22 amide and brefeldin A, indicating the involvement of GLP-1-R and PKA pathways [40]. On a molecular level, the c-Jun N-terminal kinase (JNK) signaling pathway, which mediates the pro-apoptotic effects of palmitate, was inhibited by Ex-4 pretreatment. Specifically, Ex-4 reduced the phosphorylation of *JNK1/2* and *c-Jun* [40]. These results are supported by the findings of He et al. [16], who demonstrated that treatment of hASC with 100 nM Ex-4 led to a significant reduction in Hoechst-positive cells, reduced DNA fragmentation, and fewer cells entering early stages of apoptosis. Additionally, a dose-dependent increase in the B-cell lymphoma 2 (BCL-2)/Bcl-2–associated X protein (BAX) was observed, indicating a shift toward cell survival. Ex-4 also activates phosphorylation of key signaling proteins in the MAPK, phosphoinositide 3-kinase (PI3K)/Akt, and cAMP pathways. However, this phosphorylation was blocked by the GLP-1-R-Antagonist Exendin(9-36), emphasizing the role of GLP-1-mediated signaling [16]. Ex-4 also enhances hMSC resistance to high-glucose and oxidative stress conditions by activating GLP-1–AMP-activated protein kinase (AMPK) [17, 18]. HMSC pretreated with Ex-4 or modified to express Exendin-4 (hMSC-Ex-4) showed reduced H₂O₂-induced apoptosis and increased phosphorylated AMPK levels, effects blocked by the AMPK inhibitor compound C [18]. In endothelial progenitor cells (EPC) derived from human peripheral blood mononuclear cells, high glucose conditions downregulate GLP-1-R and sirtuin 1 (*SIRT1*) expression, leading to impaired cell functions and increased apoptosis via elevated acetylated p53 levels. Ex-4 treatment restores EPC function under high-glucose conditions by upregulating SIRT1, which enhances cell survival, migration, and angiogenic capacity. This suggests that Ex-4 mediates its protective effects through a GLP-1-R-SIRT1-p53 signaling axis [41].

#### Liraglutide

Liraglutide shows similar properties and promotes osteogenic differentiation in hBMSC by activating the BMP2/Smad/Runx2 pathway visible in Western blots after BMP receptor inhibition [21]. In human dental pulp stem cells (hDPSC), the expression of the osteogenic markers *RUNX2* , *COL1*, osteonectin, and osteocalcin was upregulated via the LINC00968/miR-3658 axis [27]. Conversely, adipogenic and lipogenic markers such as *PPARγ*, *C/EBPα*,* FABP4*, *LPL*, and *SREBP1* were consistently downregulated [12, 13, 20, 31]. Additionally, during adipogenic differentiation, Liraglutide suppressed the mRNA expression of *GSK-3α/β* more effectively than insulin, despite similar protein levels. However, phosphorylated GSK-3β was significantly upregulated on day 21 in the Liraglutide group, suggesting enhanced Wnt-signaling repressing adipogenesis [20]. Similarly to Ex-4, Liraglutide could reduce signs of apoptosis, most likely through activation of the PKA/beta-catenin pathway [39]. In LPS-challenged hMSC, Liraglutide reversed the increased expression of cleaved caspase-3/−9 and restored Bcl-2 levels, as confirmed by flow cytometry, TUNEL staining, and Western blot. The anti-apoptotic effect was attenuated by PKA inhibition or GLP-1-R knockdown, confirming its dependence on GLP-1-R-PKA/β-catenin signaling. However, another study by Cantini et al., used GLP-1(7-36), GLP-1(9-36) and Liraglutide to induce apoptosis in unstressed hASC, as assessed in annexin-V expression on the surface, though only the effect of Liraglutide and GLP-1(7-36) was partially reversed by the GLP-1-R-Antagonist Exendin(9-36), supporting receptor-specific activity [12].

**Fig. 4:**
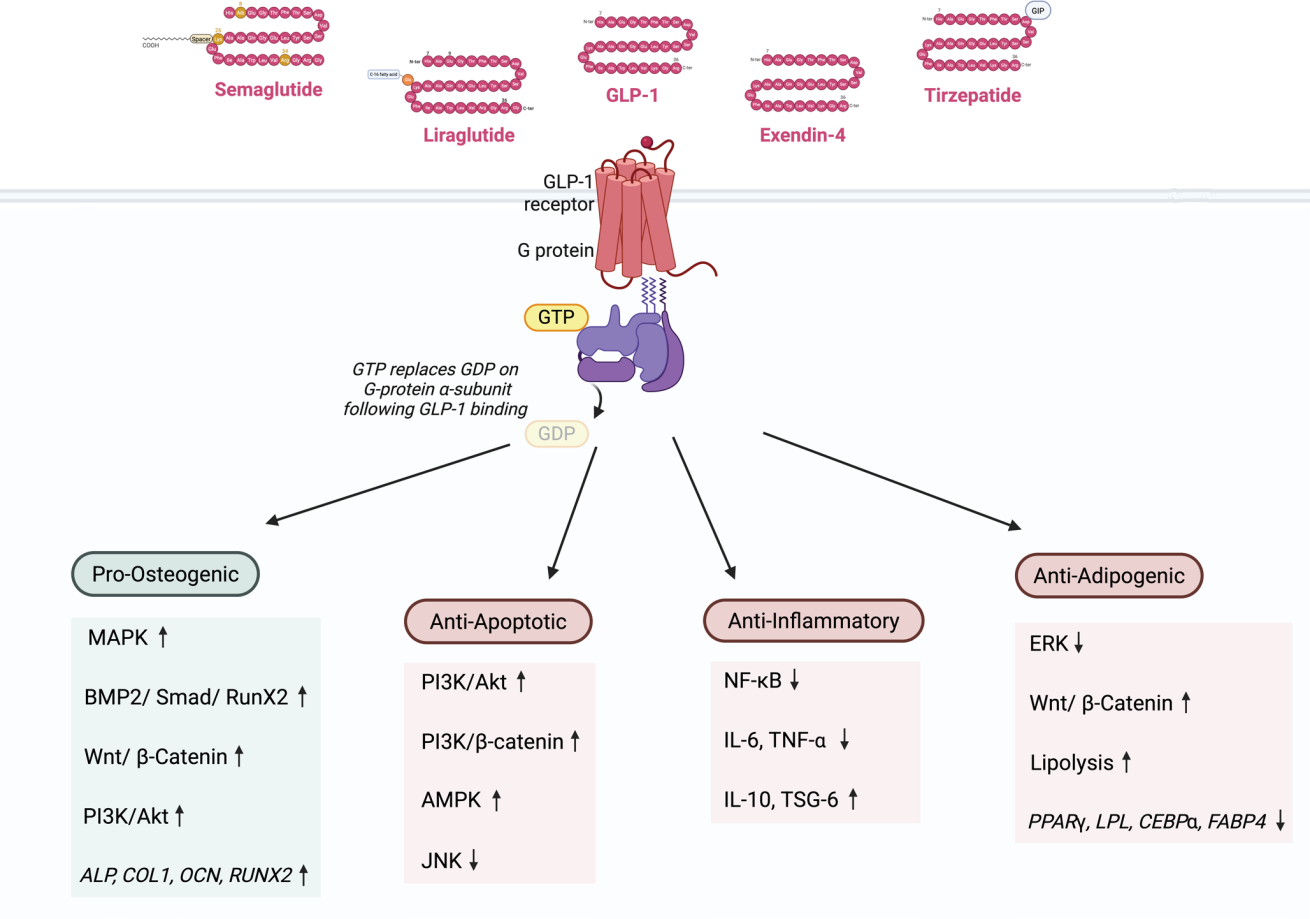
Schematic representation of intracellular signaling pathways modulated by GLP-1-RA in hMSC. Binding of GLP-1 and its analogues (Semaglutide, Liraglutide, GLP-1, Exendin-4, and Tirzepatide) to the GLP-1-R (a G protein–coupled receptor) leads to activation of downstream signaling cascades via G-protein-mediated exchange of GDP for GTP. These pathways promote pro-osteogenic effects (via MAPK, BMP2/Smad/Runx2, Wnt/β-catenin, and PI3K/Akt signaling), anti-apoptotic responses (via PI3K/Akt, PI3K/β-catenin, AMPK upregulation, and JNK inhibition), anti-inflammatory effects (via NF-κB inhibition, reduced IL-6 and TNF-α, and increased IL-10 and TSG-6), and anti-adipogenic activity (via ERK inhibition, enhanced Wnt/β-catenin signaling, increased lipolysis, and downregulation of adipogenic genes such as *PPARγ*,* LPL*,* C/EBPα*, and *FABP4*). These multifaceted effects highlight the therapeutic potential of GLP-1-RA in regenerative and metabolic medicine

GLP-1-RA such as Ex-4 and Liraglutide modulate multiple signaling pathways in hMSC and progenitor cells to enhance osteogenesis, suppress adipogenesis, promote survival, and reduce inflammation. These effects are largely mediated through activation of Wnt/β-catenin, BMP2/Smad/Runx2, PI3K/Akt, MAPK, and GLP-1R–dependent cascades, supporting their potential as multifunctional modulators in regenerative medicine (Fig. 4).

### Current Applications

#### Differentiation into Insulin-Producing Cells

Further applications for GLP1-RA, primarily Ex-4, on hMSC include the differentiation into insulin-producing cells (IPC), a process of significant interest for regenerative approaches in gastroenterology. Diseases such as diabetes continue to rise globally and impose a substantial socioeconomic burden estimated at over USD 966 billion annually [42]. In this context, the ability to differentiate abundantly available hMSC into IPC holds great therapeutic promise for future cell-based interventions.

There are a multitude of successful differentiation protocols, mostly based on a combination of cell culture medium with different growth supplements, nicotinamide and beta-mercaptoethanol with an average protocol duration of 17 days (Table 3). These protocols recapitulate key cues of pancreatic development and typically involve multistep differentiation procedures. Initial pre-induction stages utilize compounds like nicotinamide, β-mercaptoethanol, and retinoic acid, followed by exposure to high-glucose Dulbecco’s Modified Eagle Medium (DMEM) supplemented with growth factors such as activin A, hepatocyte growth factor (HGF), or epidermal growth factor (EGF). In the final maturation phase, Ex-4 or GLP-1 is introduced at concentrations ranging from 10 to 50 nM.

**Table 3:** Comparison of IPC-differentiation protocols

Source	Cell Source	Exendin-4 concentration (mM)	Differentiation protocol	Duration (days)	Additional information
Ikemoto et al. [43]	hASC	1 × 10^−5^	(1) 1% Fetal bovine serum, 1% B27 supplement, 1% N2 supplement, 50 ng/ml human activin A (2) 10 mM Nicotinamide, 50 ng/ml human hepatocyte growth factor, 1 mM histone deacetylase inhibitor	(1) 7 (2) 14	Histone deacetylase inhibitor significantly enhanced IPC differentiation (21.6 days vs. 38.8 days, *p* < 0.05), improved islet morphology score and glucose stimulation index
Ren et al. [44]	hASC	1 × 10^−5^	1) High-glucose Dulbecco’s Modified Eagle Medium (DMEM)/F12 medium, 55 nM TSA, 10 mg/l insulin (2) induced group (250 ng/ml INGAP-PP) and control group (250 ng/ml Scrambled-P) (3) 10 mM Dexamethasone, 10mM Nicotinamide (4) 10ng/ml TGF-beta1, 10 nM GLP-1, 10mM Exendin-4	(1) 3 (2) 5 (3) 5 (4) 3	(Islet Neogenesis-associated protein pentadecapeptide (INGA-PP) led to higher levels of expression and secretion of insulin and C-peptide, cell morphology and size more similar to fresh human islet cells
Silva et al. [45]	hASC	10	(1) nicotinamide, 2-Mercaptoethanol (2) additional 10 mmol/l Exendin-4	(1) 7 (2) 7	Islet-like appearance after 4 months of differentiation, cells produced insulin but not glucagon
El-Asfar et al. [35]	hASC	1 × 10^−5^	(1) 10 mmol/l nicotinamide, 1 mmol/L beta-mercaptoethanol in Low Glucose-DMEM (2) 10 mmol/L nicotinamid, 1 mmol/L β-mercaptoethanol in serum-free High Glucose-DMEM (3) 10 nmol/L exendin-4, 10 nmol/L GLP-1 or 100 nmol/L obestatin	(1) 2 (2) 1 (3) 7	IPCs generated using exendin-4 showed a significant increase in insulin secretion upon challenging with increased glucose concentration. However, the IPCs generated using either GLP-1 or obestatin, although showing a significantly increased insulin secretion as compared to exendin-4 at low glucose concentration, lacked responsiveness to increased glucose concentration
El-Asfar et al. [35]	Wharton’s jelly-derived MSC	1 × 10^−5^	(1) 5% FBS high-glucose DMEM (2) 10 mmol/L nicotinamide (3) 10 nmol/L exendin-4, 10 nmol/L GLP-1 or 100 nmol/L obestatin	(1) 14 (2) 7 (3) 7	PCs generated using either GLP-1 or obestatin showed higher secretion of insulin under low glucose conditions as compared to those derived using exendin-4. However, all of them were relatively non-responsive upon glucose challenge
Moshtagh et al. [36]	hASC	1 × 10^−5^	(1) low-glucose DMEM with 5% FBS, 0.5mmol/l β-mercaptoethanol, 10 mmol/l nicotinamide (2) high-glucose DMEM with 2.5% FBS, 0.5mmol/l β-mercaptoethanol, 10 mmol/l nicotinamide (3) high-glucose DMEM, 0.5% FBS, 0.5 mmol/l β-mercaptoethanol, 10 mmol/l nicotinamide, 10 nmol/l exendin-4	(1) 2 (2) 10 (3) 14	Expression of insulin, PDX1m Ngn3, PAX4 confirmed in rt-PCR, positive staining for dithizone, insulin production
Gao et al. [46]	umbilical cord-derived MSC	1 × 10^−5^	(1) high glucose DMEM, 10% fetal bovine serum, 10^−6^ M retinoic acid (2) high glucose DMEM, 10% fetal bovine serum (3) low glucose DMEM, 10% fetal bovine serum, 10 mmol/l nicotinamide, 20 ng/ml epidermal growth factor (4) low glucose DMEM, 10% fetal bovine serum, 10 nmol/l exendin-4	(1) 2 (2) 2 (3) 6 (4) 6	Appearance of islet-like clusters after 9 days of differentiation, synthesis and production of functional islet proteins but insulin production did not respond to glucose challenge well
Seyedi et al. [47]	umbilical cord-derived MSC	1 × 10^−5^	serum-free DMEM/F12 medium, 17.5 mM glucose, 10 mM nicotinamide, 10nM exendin-4, 10nM pentagastrin, 100pM hepatocyte growth factor, B-27, 1% penicillin/streptomycin and 1 µg/ml amphotericin B	14	Hanging drop culture showed better differentiation than monolayer: 40-fold higher insulin secretion in glucose challenge test
Santos et al. [48]	umbilical cord-derived MSC	1 × 10^−5^	(1) high-glucose 23 mmol/L, nicotinamide and 2-mercaptoethanol (2) additional 10 nmol/l exendin-4	(1) 7 (2) 7	Expressed pancreatic endocrine phenotype (islet like appearance, production of somatostatin and glucagon and expression of PDX-1) but cells did not produce insulin effectively
Nekoei et al. [49]	Wharton’s jelly-derived MSC	no information	(1) high glucose DMEMF12, 10% fetal bovine serum, 106 mol/L retinoic acid, 1% antibiotic (2) DMEMF12, 10% fetal bovine serum (3) DMEMF12, 10% fetal bovine serum, 10 mmol/L nicotinic acid, 20 ng/ml epidermal growth factor (4) DMEMF12, 10% fetal bovine serum, Exendin-4	(1) 2 (2) 2 (3) 7 (4) 7	During differentiation cells change morphology to epithelioid cells and formed three dimensional clusters, expression of PDX1, NGN3, Glut2 and insulin, glucose challenge test confirmed a significant increase in insulin secretion
Kassem et al. [37]	Wharton’s jelly-derived MSC	1 × 10^−5^	(1) low glucose DMEM, 10 mmol/l nicotinamide, 1 mmol/l beta-mercaptoethanol, 10% fetal bovine serum (2) high-glucose DMEM, 10 mmol/l nicotinamide, 1 mmol/l beta-mercaptoethanol (optional 3) high glucose-DMEM, 10 mmol/l nicotinamide, 1 mmol/l beta-mercaptoethanol, 10 nmol/l exendin-4	(1) 2 (2) 1 (3) 7	Incorporating exendin-4 enhances expression levels of Pdx-1, Nkx2.2, Isl-1 and MafA, better response to variable glucose concentrations
Xin et al. [50]	BMSC	1 × 10^−2^	(1) high glucose DMEM, 5% fetal bovine serum (2) L-DMEM, 5% fetal bovine serum, 20µmol/L nicotinamide (3) additional 10µmol/L Exendin-4	(1) 15 (2) 7 (3) 7	Dithizone staining positive, expression of pancreatic beta-cell markers, 43% of IPC showed L-type Ca^2+^-channel activity
Hosseini et al. [51]	BMSC	9.43 × 10^−3^	(1) 10% FBS/low-glucose DMEM, 1% non-essential amino acids (2) 5% FBS/high-glucose DMEM, 0.01 mol/L Nicotinamide, 20ng/ml basic fibroblast growth factor, 1mM beta-mercaptoethanol (3) 5% FBS/low-glucose DMEM, 50ng/ml Ex-4, 10ng/ml EGF, 0.01 mol/L nicotinamide	(1) 3 (2) 7 (3) 11	Cells encapsulated in Alginate differentiated more efficiently due to closeness to native ECM and higher cell-cell-contacts

Functional and phenotypic analyses confirm the induction of a pancreatic endocrine fate, including positive dithizone (DTZ) staining indicative of insulin production, as well as the upregulation of key pancreatic markers such as paired box gene 4 (PAX4), neurogenin 3 (NGN3), Isl-1, Some protocols utilized additional strategies like fibrin encapsulation, decellularized amniotic membrane [52] or 3D alginate encapsulation [51] scaffolds to enhance differentiation efficiency and mimic physiological conditions more closely. Throughout the differentiation hMSC lose their spindle-like morphology and adapt to a more epithelioid shape organized in islet-like clusters [35]. Functional and phenotypic analyses confirm the induction of a pancreatic endocrine fate, including positive dithizone (DTZ) staining indicative of insulin production, as well as the upregulation of key pancreatic markers such as *Nestin*, *PDX-1*, paired box gene 4 (*PAX4*), neurogenin 3 (*NGN3*), Isl-1, MAFA, insulin, and glucose transporter 2 (*GLUT2*). Ex-4 especially has been shown to pronounce *PDX-1* and *MAFA* gene expression, while GLP-1 failed to induce comparable expression levels [35]. Successful differentiation into IPC has been reported using hMSC derived from various tissues, including adipose tissue [35, 36, 43–45], umbilical cord [46–48], Wharton’s jelly [37, 49, 50] and bone marrow [51].

A comparative analysis by Bozorgi et al. [52] evaluated three fibrin-encapsulated hASC protocols [37, 49, 50], identifying protocol [37] as the most effective in supporting differentiation. The addition of fibrin encapsulation further enhanced differentiation efficiency and structural integrity.

Similar effects have been demonstrated in rodent MSC, where treatment with Ex at 10ng/ml ≈ 2.4 nM led to upregulation of pancreatic genes such as *PDX-1*, *GLUT2* and* Insulin* as well as an increase in DTZ-positive cells [53, 54]. Comparable results were observed with 10nM Ex-4 [55, 56], thus allowing the conclusion that there is no major species-specific difference in this context. Despite the promising results, the glucose-responsiveness of these IPC remains limited. Their insufficient insulin secretion in response to glucose stimulation highlights the need for further protocol optimization before clinical translation can be considered a feasible approach [35, 48].

#### Tenogenic Differentiation and Musculoskeletal Applications

Another potential application for differentiation enhancement lies in tenogenic differentiation. Abdulmalik et al., demonstrated a significantly increased expression of collagen and sulfated glycosaminoglycans (s-GAG), as well as the expression of tendon-specific genes, in Ex-4-treated hMSC compared to both untreated and insulin-treated controls [14].

Recent advances in biomaterial science have also enabled the incorporation of Ex-4 into novel scaffold systems using electrospinning methods. In a study utilizing Ex-4-loaded nanotube-fiber matrices, the scaffolds demonstrated excellent cytocompatibility, supporting cell attachment and proliferation regardless of whether Ex-4 was embedded within the matrix or supplied exogenously through the culture medium. Interestingly, cell count, and DNA concentration were even higher in the scaffold-based system compared to soluble Ex-4 exposure or the negative control, suggesting a superior microenvironment for cell viability and expansion. Beyond supporting cytocompatibility, these fiber matrices were capable of inducing tenogenic differentiation at levels comparable to traditional Ex-4 supplementation, as indicated by comparable deposition of collagen and s-GAG [57]. Thus, Ex-4 has also shown promising potential in promoting tenogenic differentiation of human mesenchymal stem cells, expanding its relevance to musculoskeletal regenerative applications.

#### Genetic Engineering Approaches

Genetic engineering approaches have also been employed to enhance the therapeutic potential of hMSC through the stable overexpression of Ex-4. HMSC transduced with lentiviral vectors to overexpress Ex-4 (MSC-Ex-4) maintained typical hMSC morphology, viability, and surface marker expression (CD44, CD73, CD90, and CD105), indicating that the genetic modification did not compromise stemness or core phenotypic characteristics [18, 26]. These engineered cells secreted approximately 15 ng of Ex-4 into the conditioned medium every 24 h, with the peptide released directly into the extracellular environment rather than through extracellular vesicles. Interestingly, both hMSC-Ex-4 and wild-type hMSC treated with soluble Ex-4 exhibited elevated mRNA expression of GLP-1-R in a dose-dependent manner, suggesting a feedback mechanism that may potentiate hMSC responsiveness to Ex-4 [18].

Transcriptomic analyses of MSC-Ex-4 under glucose stimulation (25 mM) revealed 1323 differentially expressed genes, with enrichment patterns indicating regulatory effects on pathways related to proliferation, apoptosis, and cell cycle regulation. Furthermore, the secretome of hMSC-Ex-4 displayed an altered protein profile, including enhanced secretion of insulin-like growth factor–binding protein 2 (IGFBP2) and apolipoprotein M (APOM), both of which are implicated in metabolic regulation and may contribute to the therapeutic modulation of type 2 diabetes mellitus [18].

#### Initial in-Vivo Investigations

Initial efforts to translate these in vitro findings into in vivo models have already been undertaken. A study by Stafeev et al. isolated hASC from diabetes mellitus type 2 patients before and after 6 months of semaglutide treatment to assess potential alteration in cell proliferation and adipogenic differentiation capacity. After treatment, hASC did not exhibit a significant change in metabolic activity as measured by MTT assay; however, the observed increased number of proliferating cell nuclear antigen (PCNA)-positive cells suggests an enhanced proliferative capacity following semaglutide therapy.

In the context of white adipogenic differentiation, commonly impaired in T2D, semaglutide increased adipocyte numbers and induced a shift toward a more lipolytic phenotype, as indicated by a reduction in medium-sized lipid droplets. This was accompanied by upregulation of *FABP4* and *HSL*, alongside reduced receptor for advanced glycation end products (*RAGE*) expression, suggesting enhanced lipid turnover and improved adipocyte function. Although the number of beige adipocytes did not increase, they displayed a more thermogenic phenotype characterized by smaller LDs, elevated expression of uncoupling protein 1 (*UCP1*) and *HSL*, and enhanced glucose utilization, all features consistent with increased mitochondrial uncoupling and browning.

These findings should be interpreted with caution, since this study is subject to potential confounding, as lifestyle modifications during the 6-month period, such as changes in diet, physical activity, or medication adherence, may have influenced stem cell characteristics independently of semaglutide treatment [58].

Future applications of GLP-1-RA may extend beyond current differentiation strategies, potentially enabling targeted tissue-specific modulation through biomaterial integration, gene editing, or combination therapies. Ongoing comparative in vitro studies aim to systematically evaluate and distinguish the specific effects of different GLP-1-RA on hMSC behavior, paving the way for optimized, indication-specific regenerative approaches.

##  Conclusion

This systematic review highlights the significant impact of GLP-1-RA on hMSC differentiation and function, mediated through key pathways such as Wnt/β-catenin, BMP/Smad, PI3K/Akt, and PKA. Both Ex-4 and Liraglutide were shown to enhance osteogenesis, suppress adipogenesis, and improve cell viability under stress conditions like hyperglycemia or inflammation. As most of the available evidence stems from in vitro and preclinical studies, there remains a need for long-term and in vivo research to substantiate these findings and assess their translational relevance. Furthermore, hMSC represent a heterogeneous population with cells derived from different tissue sources exhibiting different biological properties. Despite promising findings, the lack of standardized experimental models and inconsistent GLP-1-RA dosing regimens across studies limits direct comparison and reproducibility. Future research should aim to harmonize differentiation protocols, stem cell sources, and treatment concentrations to better define the therapeutic window and mechanisms of GLP-1-RA in regenerative applications.

## Data Availability

No datasets were generated or analysed during the current study.

## Abbreviations

ADIPOQ: Adiponectin
ADRP: Adipose differentiation-related protein
ADSC: Adipose tissue derived Stem Cells
ALP: Alkaline phosphatase
AMPK: AMP-activated protein kinase
ANG-1: Angiopoietin-1
APN: Adiponectin
APOM: Apolipoprotein M
AP2: Activator Protein 2
ATGL: Adipose triglyceride lipase
BAX: Bcl-2-associated X protein
BCL-2: B-cell lymphoma 2
BMP2: Bone morphogenetic protein 2
BMSC: Bone marrow mesenchymal stem cell
C/EBPα: CCAAT/enhancer-binding protein alpha
cAMP: Cyclic adenosine monophosphate
CAV1: Caveolin-1
CCK-8: Cell Counting Kit-8
CDK: Cyclin-dependent kinase
CERSS: Ceramide synthase 5
COL1: Collagen type I
COL10A1: Collagen type X alpha 1 chain
COL1A1: Collagen type I alpha 1 chain
COL2: Collagen type II
COLI: Collagen-1
DEGS1: Delta (4) -desaturase, sphingolipid 1
DKK-1: Dickkopf-1
DLK1: Delta-like 1 homolog
DMEM: Dulbecco’s Modified Eagle Medium
DTZ: Dithizone
EAT: Epicardial adipose tissue
EdU: 5-ethynyl-2′-deoxyuridin
EGF: Endothelial-growth factor
EPC: Endothelial progenitor cells
ERK: Extracellular signal-regulated kinase
Ex-4: Exendin-4
FABP4: Fatty acid-binding protein 4
FASN: Fatty acid synthase
FBS: Fetal bovine serum
FOXO1: Forkhead box protein O1
GIP: Gastric inhibitory polypeptide
GIP-R: Gastric inhibitory peptide receptor
GIP-RA: Gastric inhibitory polypeptide receptor agonist
GLP-1: Glucagon-like peptide-1
GLP-1-R: Glucagon-like peptide-1 receptor
GLP-1-RA: Glucagon-like-peptide-1 receptor agonist
GLUT1: Glucose transporter type 1
GLUT2: Glucose transporter type 2
GLUT4: Glucose transporter type 4
GSK-3β: Glycogensynthase-kinase 3 beta
hASC: human adipose-derived stem cells
hDPSC: Human dental pulp stem cells
HGF: Hepatocyte-growth factor
hMSC: Human mesenchymal stem cells
HMSC-E4: HMSC that overexpress Ex-4
HSL: Hormone-sensitive lipase
IBMX: 3-Isobutyl-1-methylxanthine
IGFBP2: Insulin-like growth factor-binding protein 2
IL-10: Interleukin 10
IL1B: Interleukin 1 beta
IL6: Interleukin 6
INGA-PP: Islet Neogenesis-associated protein pentadecapeptide
INSR: Insulin receptor
IPC: Insulin-producing cells
ISL-1: ISL LIM homeobox 1
JNK: c-Jun N-terminal kinase
KGF: Keratinocyte growth factor
LD: Lipid droplets
LiCl: Lithium chloride
LINC000968: Long intergenic non-coding RNA 968
LPL: Lipoprotein lipase
LPS: Lipopolysaccharides
MAFA: V-maf musculoaponeurotic fibrosarcoma oncogene homolog A
MAPK: Mitogen-activated protein kinase
MCP1: Monocyte chemoattractant protein-1
miRNA: MicroRNA
MMP9: Matrix-Metalloproteinase 9
MTS: 3-(4,5-Dimethylthiazol-2-yl) -5-(3-carboxymethoxyphenyl) -2-(4-sulfophenyl) -2 H-tetrazolium
MTT: 3-(4,5-di methylthiazol-2-yl) -2,5-diphenyltetrazolium bromide
NFkB: Nuclear factor kappa-light-chain-enhancer of activated B cells
NGN3: Neurogenin-3
Nkx2.2: NK2 homeobox 2
OCN: Osteocalcin
OSX: Osterix (SP7 transcription factor)
PAX4: Paired box gene 4
PCNA: Proliferating cell nuclear antigen
PDLSC: Periodontal ligament stem cells
PDX-1: Pancreatic and duodenal homeobox 1
PGC1α: Peroxisome proliferator-activated receptor gamma coactivator 1-alpha
PI3K: Phosphoinositide 3-kinase
PKA: Protein kinase A
PLIN1: Perilipin 1
PPARγ: Peroxisome proliferator-activated receptor gamma
pref-1: Pre-adipocyte factor-1
QUIN: Quality Assessment Tool For In Vitro Studies
RAGE: Receptor for advanced glycation end products
RUNX2: Runt-related transcription factor 2
s-GAG: sulfated glycosaminoglycans
SAT: Subcutaneous adipose tissue
SDF-1: Stromal cell-derived factor 1
SIRT1: Sirtuin 1
SOX2: SRY-box transcription factor 2
SPC: Surfactant protein C
sPLA2: Secretory phospholipase A₂
SREBP1: Sterol regulatory element-binding protein 1
SVF: Stromal vascular fraction
T2D: type 2 diabetes
TNF- α: Tumor necrosis factor alpha
TSG-6: Tumor necrosis factor-stimulated gene 6
UCP1: Uncoupling protein 1
VAT: Visceral adipose tissue
VDAC3: Voltage-dependent Anion Channel 3
Wnt: Wingless/Integrated signaling pathway
WNT3A: Wnt family member 3 A
αMEM: α-Minimum Essential Medium
